# Development and assessment of an active strategy for the implementation of a collaborative care approach for depression in primary care (the INDI·i project)

**DOI:** 10.1186/s12913-017-2774-2

**Published:** 2017-12-13

**Authors:** Enric Aragonès, Diego Palao, Germán López-Cortacans, Antonia Caballero, Narcís Cardoner, Pilar Casaus, Myriam Cavero, José Antonio Monreal, Víctor Pérez-Sola, Miquel Cirera, Maite Loren, Eva Bellerino, Catarina Tomé-Pires, Laura Palacios

**Affiliations:** 1 Primary Care Area Camp de Tarragona, Catalan Health Institute, Tarragona, Spain; 2 Primary Care Research Institute IDIAP Jordi Gol, Barcelona, Spain; 3 Mental Health Service, University Hospital Parc Taulí, Sabadell, Spain; 4 Department of Psychiatry and Legal Medicine, Autonomous University of Barcelona, Barcelona, Spain; 5 University Psychiatric Hospital Pere Mata Institute, Reus, Spain; 6 Mental Health Centre Esquerra Eixample, Hospital Clínic, Barcelona, Spain; 7 Institute of Neuropsychiatry and Addictions, Hospital del Mar, IMIM, Barcelona, Spain; 8 CIBERSAM, Madrid, Spain; 9 Healthcare Corporation Parc Taulí, Primary Care Area, Sabadell, Spain; 10 Primary Care Service Vallès Occidental, Catalan Health Institute, Sabadell, Spain; 11 Unit for the Study and Treatment of Pain – ALGOS, Universitat Rovira i Virgili, Tarragona, Spain; 12 Department of Psychology, Universitat Rovira i Virgili, Tarragona, Spain; 13 Centre d’Atenció Primària de Constantí, Carrer dels Horts, 6, 43120 Constantí (Tarragona), Spain

**Keywords:** Depressive disorder, Primary health care, Health plan implementation, Disease management, Patient-centered care

## Abstract

**Background:**

Primary care is the principal clinical setting for the management of depression. However, significant shortcomings have been detected in its diagnosis and clinical management, as well as in patient outcomes. We developed the INDI collaborative care model to improve the management of depression in primary care. This intervention has been favorably evaluated in terms of clinical efficacy and cost-effectiveness in a clinical trial. Our aim is to bring this intervention from the scientific context into clinical practice.

**Methods:**

*Objective:* To test for the feasibility and impact of a strategy for implementing the INDI model for depression in primary care.

*Design:* A quasi-experiment conducted in primary care. Several areas will be established to implement the new program and other, comparable areas will serve as control group. The study constitutes the preliminary phase preceding generalization of the model in the Catalan public healthcare system.

*Participants:* The target population of the intervention are patients with major depression. The implementation strategy will also involve healthcare professionals, primary care centers, as well as management departments and the healthcare organization itself in the geographical areas where the study will be conducted: Camp de Tarragona and Vallès Occidental (Catalonia).

*Intervention:* The INDI model is a program for improving the management of depression involving clinical, instructional, and organizational interventions including the participation of nurses as care managers, the efficacy and efficiency of which has been proven in a clinical trial. We will design an active implementation strategy for this model based on the PARIHS (Promoting Action on Research Implementation in Health Services) framework.

*Measures:* Qualitative and quantitative measures will be used to evaluate variables related to the successful implementation of the model: acceptability, utility, penetration, sustainability, and clinical impact.

**Discussion:**

This project tests the transferability of a healthcare intervention supported by scientific research to clinical practice. If implementation is successful in this experimental phase, we will use the information and experience obtained to propose and plan the generalization of the INDI model for depression in the Catalan healthcare system. We expect the program to benefit patients, the healthcare system, and society.

**Trial registration:**

ClinicalTrials.gov identifier: NCT03285659; Registered 12th September, 2017.

## Background

Major depression is a highly prevalent disorder. The ESEMED study found the lifetime prevalence of major depressive disorder to be 10.6% in Spain, and 12-month prevalence to be 4% [1]. Depression is an undeniable public health problem, impacting not only patients and those around them but society as a whole [2]. In 2006, the cost of depression in Catalonia was 735 million euros. Direct healthcare costs represented only 21% of this total, while the majority were indirect costs related to the loss of labor productivity, inability to work, and premature death by suicide [3].

The most common mental health disorders, including depression, are managed in a primary care context [4]. A study conducted in primary care centers in Catalonia found that 14% of consecutive patients seen for any reason met criteria for major depression [5], and there is general consensus that the level of care at which depression can be managed most adequately and efficiently is primary care, in both developing and developed countries [6].

However, there are inadequacies in diagnosis, treatment, and follow-up for patients with this disorder [7], and clinical outcomes are frequently unsatisfactory [8]. In primary care, the monitoring of patients with depression is rarely planned, and there is inadequate supervision of clinical progress and adherence to treatment. Therefore, opportunities to take measures to increase adherence or adjust treatment when progress is not satisfactory are often missed [9].

In the management of depression there is a gap between what works and what actually happens in clinical practice and, because of this, clinical outcomes for depression do not correspond to what might be expected based on the efficacy of therapeutic interventions [10]. Studies have shown that collaborative care models are an appropriate strategy for closing this gap and achieving better health outcomes by bringing depression management closer to effective recommendations based on scientific evidence [11].

Collaborative care models are complex care programs based on the chronic care model and, in terms of structure, they encompass several components [12]. They are shared care models in which the roles of the various professionals involved in the management of depression are integrated into a common structure. The primary care physician is responsible for the clinical process of diagnosing and treating patients with depression. The care manager (often a nurse) assists the family physician in proactively following up on the patient’s clinical progress and adherence to treatment, and is the main person responsible for fostering patient empowerment and active participation in the therapeutic process. Psychiatrists assume different levels of involvement in different models, but in every model their role is to supervise and assist the primary care professionals in depression management, especially in cases with a less than satisfactory response or other complicating factors. Empowered and active patients, and by extension their family members, are generally considered to be part of the therapeutic team. A key aspect of collaborative care models is the systematic monitoring of the patient’s clinical progress and therapeutic response, which makes it possible to determine the most appropriate therapeutic approach for each patient’s clinical situation at any given time.

The efficacy of collaborative care models in the treatment of depression has been demonstrated in more than 70 clinical trials systematized in several meta-analyses [13]. Thota’s meta-analysis [14] evaluated 32 randomized clinical trials, and reported a standardized mean difference of 0.34, which, although conventionally interpreted as a small to moderate effect, is considered relevant in clinical and public health contexts given the prevalence and impact of depression. In general, it was concluded that there is sufficient scientific evidence to recommend the widespread implementation of these clinical models, which are especially applicable in the primary care setting [15].

Although the first models were developed and evaluated beginning 20 years ago almost exclusively in the United States [11], in recent years several clinical trials have demonstrated the efficacy, feasibility, and utility of these models in European public healthcare systems [16]. One of these studies is the INDI project, the basis for this project, which has developed and evaluated a collaborative care model adapted to the Catalan healthcare system [17].

Therefore, scientific evidence supports the clinical efficacy of collaborative care models for improving clinical results in depression, but although some useful cases have been reported [18], examples of the application or continued use of these interventions outside the research context are rare. A central challenge for healthcare systems is the implementation and generalization of health interventions that have been developed and evaluated in a research setting [19].

We aim to develop an effective strategy for implementing and generalizing the INDI model for depression management in the primary care context of the Catalan public healthcare system that is practical, effective, acceptable and perceived as useful by patients, healthcare professionals, and the organization. Our hypothesis is that the implementation of this method will give rise to the development of clinical processes that are better aligned with scientific evidence, which will result in clinical benefits for patients, and will prove economically sustainable and tenable over time.

## Methods/design

### Aim

The aim of this project is to develop a feasible strategy for the implementation of the INDI model under real healthcare conditions of primary care, initially in a limited geographical area but with the purpose of promoting the generalization of this model in the Catalan public healthcare system.

We will evaluate the success of the implementation based on its perceived acceptability and utility (to patients, professionals, and the organization) and on the integration and sustainability of the program into healthcare practice. The effectiveness of implementation will be assessed in terms of: (a) the quality and precision of the diagnosis of major depression; (b) suicide risk management; (c) the adaptation of the therapeutic management of depression to recommendations based on scientific evidence, including initial treatment selection, treatment duration, and joint management with psychiatry; (d) clinical results in depressed patients; and (e) financial costs associated with implementation.

### Design

This is a study of the implementation of a healthcare intervention with a quasi-experimental design and mixed measurement methods that include both qualitative techniques and analysis and quantitative indicators.

The design involves defining several geographic areas in which the INDI program will be implemented as well as other comparable areas which will serve as the control, where depression will be treated as usual.

### Settings and participants

This project will be completed in a limited area as a preliminary step to its eventual generalization.

**Fig. 1:**
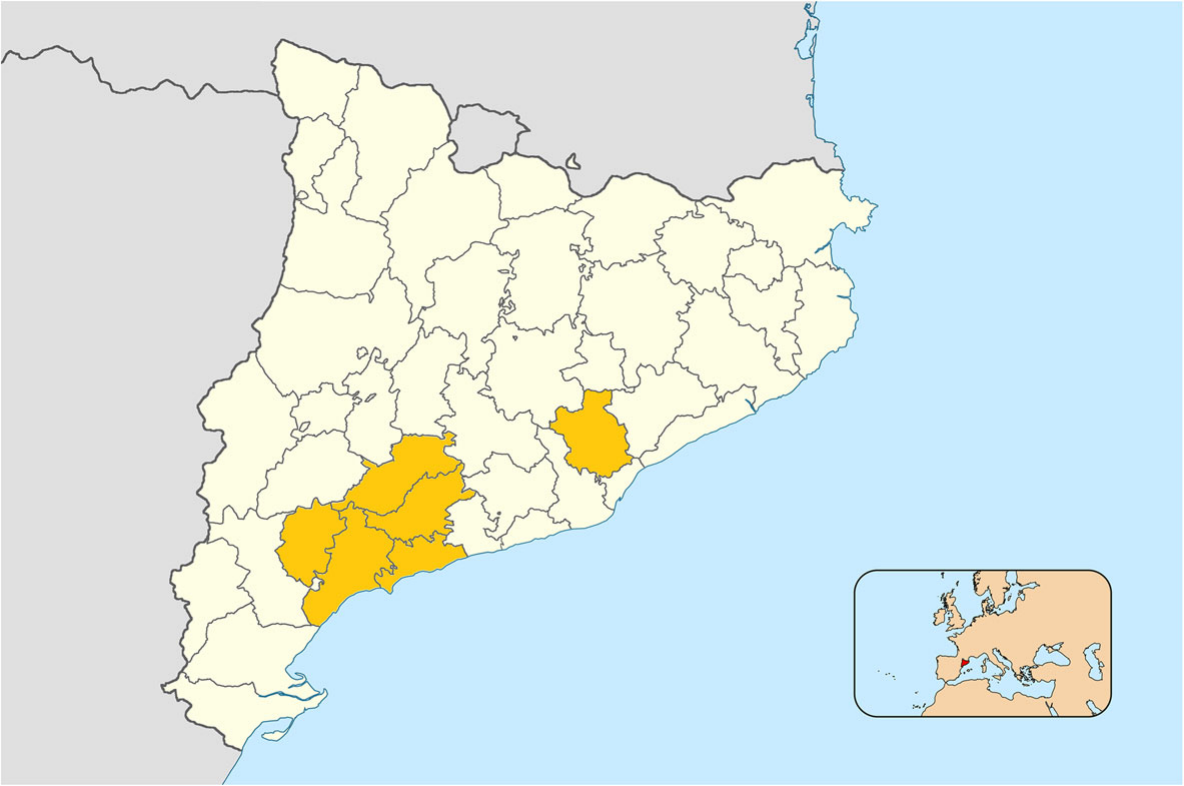
Counties of Catalonia where the study will be carried out. Modified from Martí8888 [CC BY-SA 4.0]. Available at: https://commons.wikimedia.org/wiki/File:Catalonia_base_map_42_comarques.png

Two public healthcare administrative organizations, the Gerència Territorial Camp de Tarragona (Catalan Health Institute) and the Servei d’Atenció Primària Vallès Occidental Est (Catalan Health Institute), will participate in this phase along with the primary care centers and mental healthcare providers in both regions (Fig. 1).

The Gerència Territorial Camp de Tarragona is the main primary care provider in the counties of Alt Camp, Conca de Barberà, Baix Camp, el Tarragonès and el Priorat in southeastern Catalonia and manages 20 primary care centers serving an assigned population of 323,740 people. The entity providing mental healthcare services is the Institut Pere Mata, with a community network including three adult mental healthcare centers located in the cities of Reus, Tarragona, and Valls.

The Servei d’Atenció Primària Vallès Occidental Est is the primary care provider for part of the county of Vallès Occidental, located in the Barcelona metropolitan area, and it manages 22 primary care teams serving an assigned population of 386,811 people. The Parc Taulí Corporation is the mental healthcare services provider and has two adult mental healthcare centers located in the city of Sabadell. This entity also manages a primary care center located in Sabadell that is participating in the project.

In each of these two regions, an area for testing the intervention and a comparable control area will be selected. The assignment of an experimental or control condition will not be random, but we will ensure that the areas are comparable in their sociodemographic, health, and organizational characteristics. Because the aim of the study is to test implementation under real conditions and we primarily anticipate using intention-to-treat analyses, exclusion criteria have not been established for centers or professionals as long as they are within the areas defined for this experiment.

All adult patients with a new diagnosis of major depression or a new episode of antidepressant treatment, defined as a new prescription (with no prior treatment for at least three months) associated with a prior diagnosis, will be considered for analysis. Patients with a psychotic disorder, bipolar disorder, a disorder associated with drug or alcohol use, dementia, mental retardation, postpartum depression, institutionalized patients and patients receiving treatment in at-home care programs will be excluded from the analysis because they are not part of the target population of the intervention. However, comorbidity of depression and other common mental disorders such as anxiety or sleep disorders do not constitute exclusion criteria.

### Intervention (INDI program)

INDI is a multi-component program based on the chronic care model and previous collaborative care programs adapted to the conditions of primary care in the Catalan public healthcare system. It is made up of training, clinical and organizational components, as well as health education for patients. Its objective is to improve clinical management and clinical outcomes in depression. The program focuses on how the approach to depression is managed by the primary care team, and introduces the role of the care manager, enhances the relationship between different levels of care, strengthens the skills of the healthcare professionals involved, and empowers patients. It is based on the optimization of available resources rather than the need for additional resources. The characteristics of this program have been published previously [17, 20].

The INDI model has been tested in a clinical trial in which its clinical efficacy was found to be comparable to that reported in the scientific literature for similar interventions, with an effect size of 0.35 compared to standard treatment and depression response and remission rates 15–20% greater during 12 months of follow-up [17]. In a 3-year analysis, an attenuation of the effect parallel to the decrease in the adherence of professionals to the INDI program was observed, in the absence of an active strategy to promote the sustainability of the program [21].

In addition to clinical effectiveness, economic impact is a considerable factor in the decision to implement a healthcare intervention. The evaluation of this parameter showed an incremental cost-effectiveness ratio of €4056/quality-adjusted life year (QALY) or €4.5/depression-free day. This means that INDI yields better results than standard treatment for a modest increase in cost, which translates into a favorable cost-effectiveness ratio according to commonly accepted criteria in the field of healthcare innovation [22].

Although it was not part of the original INDI model, the current model incorporates a new tool for optimizing the clinical management of major depression. This is an interactive clinical guideline integrated in the computerized primary care clinical records. It has been developed based on the most recent clinical practice guidelines for major depression [23, 24] It assists physicians with decisions regarding diagnosis, treatment, and monitoring of major depression; systems for recording and retrieving information on a patient’s clinical status; and automated alerts for clinical situations showing poor control of the illness or risk factors [25, 26].

### Intervention (implementation strategy)

**Fig. 2:**
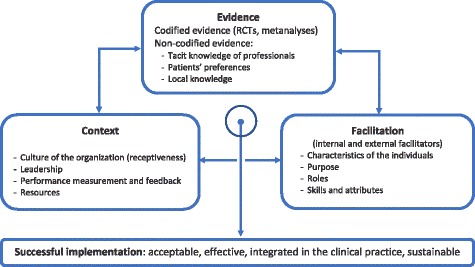
Conceptual framework for the implementation project (adapted from the PARIHS framework)

We have designed an implementation strategy conceptually based on and framed within the PARIHS model (Promoting Action on Research Implementation in Health Services) [27] (Fig. 2). This theoretical and operational framework defines implementation success as a function of a) evidence that supports the proposed innovation, b) the context in which the change is to be applied, and c) the components of the facilitation that will drive and maintain the change.

$$ \mathsf{Successful}\kern0.5em \mathsf{implementation}=\mathsf{f}\ \left(\mathsf{Evidence},\mathsf{Context},\mathsf{Facilitation}\right) $$

The PARIHS framework considers evidence that supports the innovation in a broad sense, encompassing both obvious sources of evidence (i.e., clinical trials, meta-analyses) and tacit evidence from other sources such as the experience, knowledge, and reflections of healthcare professionals who work in the field (and who, ultimately, apply the proposed changes). The needs, opinions, and preferences of the patients who the intervention is intended for are also included as sources of evidence, as well as knowledge of local circumstances and determinants. This broad understanding of evidence with the inclusion of all the project’s stakeholders is necessary to successfully implement lasting changes in healthcare practice.

Existing evidence regarding the effectiveness of collaborative care models and the INDI model in particular is abundant and solid [11, 14, 16, 17]. Through qualitative techniques such as focus groups and in-depth interviews with local leaders in the fields of primary care, mental health, and healthcare innovation, we will obtain and incorporate the tacit knowledge of patients and professionals as well as information on local characteristics into the INDI model.

The context is the setting in which the proposed change will be implemented. Context includes characteristics of the health organization itself (i.e., receptivity, culture of innovation, leadership, standard procedures for evaluating tasks and results, available resources) that favor effective implementation, as well as obstacles that the implementing team should investigate, identify, and manage. One aspect of the context that is extremely favorable to the implementation of the INDI program is that the Catalan health Institute, through the Innòbics program, fosters a culture of innovation and has taken on the implementation of INDI as a priority project.

Facilitation describes the type of support needed to help people change their attitudes, habits, skills, and ways of thinking and working. Facilitators help people understand what they should change and how to change it to achieve the desired outcome. Internal facilitators (in the health organization itself) will be designated as program leaders in each participating center or as regional project leaders (in each of the two participating regions) who will lead the project implementation at the local level.

External facilitation actions will be conducted by the implementing team, including technical support, training, advice, evaluation, feedback, adaptation of the intervention to the local context, accreditation and re-accreditation of centers and professionals, and inter-institutional coordination.

### Control

The centers in the control group will not establish any special treatment practices, but the organization and professionals will offer patients with depression the best care based on standard criteria. As this is a study of implementation under real conditions the centers in the control group will not be prevented from accessing or applying any other initiative for training or healthcare quality improvement that exists in the study period.

### Measurements

We will assess a series of variables and parameters related to the success of the implementation of the intervention in the care practice principally in terms of acceptability (i.e., the perception among patients and providers that the INDI model is agreeable, satisfactory, or useful), effectiveness (i.e., producing better clinical outcomes), penetration (i.e., the INDI model can be successfully used and become integrated within the service setting), and sustainability (i.e., the extent to which the INDI model is maintained within the service setting’s ongoing, stable procedures), and its impact both in the clinical setting and in the health organization through qualitative procedures and quantitative methods [28].

#### Qualitative assessment

Qualitative assessments will provide information on qualitative aspects of the implementation process, its impact on the health organization, and its effects on professionals and patients (Table 1).

**Table 1:** Battery of instruments and procedures for the qualitative evaluation of the perceived impact of INDI model implementation in the healthcare organization, among healthcare professionals, and with patients

Scope of assessment	Instrument	Domains	Source	Time
Healthcare organizations	ARCHO (Assessment of Readiness for Chronicity in Health Care Organizations instrument)	Implementation of chronic care models	Clinical management and leaders	Baseline and 12 months
	CPCQ (Change Process Capability Questionnaire)	Strategies used and capacities for change	Clinical management and leaders	Baseline and 12 months
Professionals	Focus groups	Acceptability, perceived utility, pervasiveness in clinical practice, sustainability, difficulties, and areas for improvement	Primary care doctors and care managers	12 months
Patients	IEXPAC (Instrument for Evaluating Patient Experience of Chronic Illness Care)	Experience of the patient with chronic illness	INDI program patients	12 months
	PACIC-D (Patient Assessment of Care for Chronic Conditions-Depression)	Alignment of the care received with the chronic care model and patient-centered care	INDI program patients	12 months
	Focus groups	Acceptability, perceived utility, pervasiveness in clinical practice, difficulties, and areas for improvement	INDI program patients	12 months

##### Qualitative evaluation at the healthcare organization level

ARCHO (Assessment of Readiness for Chronicity in Health Care Organizations) [29] is an instrument for assessing the degree of implementation of chronic care models in healthcare organizations. It is a questionnaire that contributes information for the evaluation of several dimensions: organization, community orientation, care model, patient self-care, support for clinical decision-making, and information systems. In this project, we will use a modified version for depression, which will be completed by regional management departments and primary care centers, as well as by local leaders in chronic disease management and mental health for evaluation at the meso-level (management of organizations, centers, and care programs).

The CPCQ (Change Process Capability Questionnaire) [30] is a questionnaire consisting of 32 items grouped into two domains: the first evaluates the strategies used (and their success or failure), and the second assesses the capacity for change. This instrument will allow us to determine these parameters in relation to the INDI implementation plan as well as aspects of the organizational culture.

##### Evaluation at healthcare professional level

We will conduct focus group research [31] with groups of professionals directly involved in the implementation of the care model: family physicians and primary care nurses (acting as care managers). The objectives of these groups are to evaluate concepts such as program acceptability, perceived utility, and degree of pervasiveness in clinical practice, and to identify difficulties and areas for improvement in the implementation of the model.

There will be a minimum of two focus groups for each professional tier – primary care physicians and nurses (care managers) – and they will continue until enough information is obtained.

##### Evaluation of patient opinions

The patient experience is an extremely valuable indicator in assessing the quality and utility of a health intervention. In a random sample of the depressed patients treated in the INDI program, we will use two questionnaires that yield complementary information:

The IEXPAC scale (Instrument for Evaluating Patient Experience of Chronic Illness Care) [32] measures the experience of patients with chronic diseases, in this case depression, in their interaction with healthcare professionals and services. It assesses the quality of care and the adoption of patient-centered approaches to care.

The PACIC (Patient Assessment of Chronic Illness Care) [33, 34] is a validated instrument in which patients evaluate the extent to which the care they receive is aligned with the chronic care model and patient-centered care. We will use a modified version for depression to assess patients’ experiences as recipients and active users of INDI interventions. We will also obtain information through focus groups. There will be a minimum of two focus groups, which will continue until enough information is obtained.

#### Quantitative evaluation

To measure the impact of the implementation of the model, we will evaluate a battery of quantitatively measurable indicators covering different aspects of quality, performance of the care process, and clinical outcomes: (a) diagnosis and initial evaluation (b) treatment, (c) clinical follow-up, (d) clinical outcomes, and (e) epidemiological indicators (Table 2).

**Table 2:** Battery of indicators for the quantitative evaluation of the impact of INDI model implementation on clinical processes and outcomes

Area of evaluation	Indicator	Description
Diagnosis and evaluation	Diagnostic accuracy	The diagnosis of depression in the target population includes specifying the severity of the depressive episode (mild, moderate, severe, or currently in remission) as well as whether it is a single or recurrent episode.
	Diagnostic reliability	DSM-V criteria were used when making the diagnosis (MINI interview)
	Baseline evaluation of severity	In the baseline assessment the severity of symptoms was examined with a validated scale (PHQ9)
	Baseline evaluation of suicide risk	In the baseline assessment the risk of suicide was examined with a validated scale (MINI)
Treatment	Adequacy for mild depression	In mild major depression an antidepressant is not prescribed in the first eight weeks
	Adequacy for moderate or severe depression	In moderate or severe major depression treatment with antidepressants is initiated
	Adequacy for anxiolytic treatment in depression	The prescription of an anxiolytic as the only form of treatment for depression is avoided (i.e., without an antidepressant)
	Adherence to treatment, acute phase	12 weeks after initiating treatment the prescription has not been interrupted
	Adherence to treatment, continuation phase, relapse prevention	6 months after initiating treatment the prescription has not been interrupted
	Intensification of antidepressant treatment: switch	Proportion of new treatments in which the antidepressant is changed
	Intensification of antidepressant treatment: augmentation	Proportion of new treatments in which an antidepressant and an atypical antipsychotic or lithium are concomitantly prescribed
	Intensification of anti-depressant treatment: combination	Proportion of new treatments in which two antidepressants are prescribed simultaneously
Follow-up	Use of a validated scale	Number of times per patient in which a validated scale is used (PHQ9) in clinical follow-up
	Follow-up, after initiating treatment	The patient attends at least one follow-up visit in person in the month following the initiation of antidepressant treatment
	Follow-up, acute phase	The patient attends at least three follow-up visits in the 12 weeks following the initiation of antidepressant treatment
Clinical outcomes	Evolution of the symptoms of depression	PHQ9 descriptive parameters of evolution
	Rate of response to treatment	Proportion of patients who show a reduction ≥50% in their baseline PHQ9 score at 6 and 12 months
	Rate of remission	Proportion of patients with a PHQ9 score < 5 points at 6 and 12 months
	Evolution of functional impact	The descriptive parameter of evolution of GAF scale
	Evolution of suicide risk	Descriptive parameter of the evolution of suicide risk score measured with the MINI suicide risk scale
Epidemiological indicators	Prevalence	Proportion of patients diagnosed with major depression in the population served
	Incidence	New diagnoses of major depression in the population served (annually)
	Rate of antidepressant treatment	Proportion of patients with major depression who receive antidepressant treatment

These indicators will be obtained from the SIDIAP database (Information System for Research in Primary Care) [35], which draws on information from computerized primary care records and other complementary sources.

### Analyses

The principal analyses will be by intention-to-treat, regardless of the degree of application and adherence to the INDI program guidelines by the patients, professionals, and healthcare centers or areas.

The quantitative analyses of this study will be based on the clinical records of all eligible depressed patients seen in the participating primary care centers. That is, we will work with the entire population and not with a sample, which provides the strengths to minimize selection bias and is based on an independent data collection [36].

At baseline, we will perform a comparative analysis between the areas assigned to the experimental group and the areas of the control group to ensure similarity between the characteristics of their health organizations and clinical practices related to depression.

Analyses of the quantitative variables will consist of both pre-post and intervention-control comparisons of the sets of indicators with quantitative results, with key control points at baseline and at 12 months.

Based on the nature and characteristics of the various questionnaires described in the measures section, the analyses will be carried out in a pre-post comparison framework or as a retrospective analysis at an advanced stage of the implementation process, which we have set at 12 months.

The interviews with focus groups will be recorded and transcribed in their entirety. We will then use a thematic framework analysis to classify and organize the data according to key topics, concepts, and predefined constructs that will be analyzed using qualitative techniques adapted from the normalization process theory [37] to identify barriers and facilitators in the various domains, focusing on ‘hot spots’, for example, dilemmas, conflict situations, and uncertainties.

## Trial status and forecast execution dates

This study was registered on September 12th, 2017 in *ClinicalTrials.gov* with the *Identifier: NCT03285659* (https://clinicaltrials.gov/show/NCT03285659). Design and preparation of the implementation strategy: June–December 2017; start of implementation: January 2018; implementation procedures: January 2018–June 2019; analysis of the experience, diffusion and publication of the results, design of a proposal for the generalization of the model in primary care centers in the Catalan public health system: June 2019–December 2019.

According the schedule of the trial, we are currently designing and preparing the implementation strategy and collecting pre-intervention data. Procedures for the implementation of the INDI program will be initiated in the selected healthcare regions and primary care centers in 2018.

## Discussion

Depression is a highly prevalent health problem, and has a substantial impact on individuals and society in terms of morbidity and financial cost [2]. It is the mental health disorder most closely associated with suicide [38]. Its clinical management is rooted mainly in primary care, where depression is an everyday problem, and it is also the most frequent reason for referral and shared care between primary care and psychiatry.

This project is an example of translational research: It seeks to bridge the gap between scientific knowledge and clinical practice in the management of depression, a gap that compromises clinical outcomes. The INDI model is an innovative intervention that has been shown in a clinical trial to improve the results of depression management when compared with standard management practices [20, 22]. The challenge is to translate and generalize this apparently useful and effective model, supported by scientific evidence, to healthcare practice under the real-life conditions of primary care.

This proposal has been developed in keeping with the PARIHS framework [27] which constitutes both a consistent theoretical framework and a useful operational structure for establishing and carrying out the implementation strategy, providing the whole project with scientific and methodological integrity.

However, it is important to recognize the limitations of this project. First, the effect size of the INDI model – and collaborative care models in general – in improving the clinical outcomes of depression is small to moderate. Nevertheless, considering the prevalence and burden of disease that depression represents in society, and in primary care in particular, even this moderate effect could translate into significant benefits not only for individuals with depression, but also for the healthcare system and for society as a whole [15]. Second, it has been established that without measures to maintain the involvement of the professionals and the activities of the INDI program, its performance and its beneficial effects decrease over time [21]. For this reason, our implementation strategy should be seen not as an isolated intervention for initiating the program but as a continuous process. Third, the INDI model is intended exclusively for the management of major depression, although it is commonplace in everyday practice to encounter patients with poorly defined depressive states that do not meet the diagnostic criteria or situations of comorbidity of depression with other common mental disorders [39] – particularly anxiety disorders – or chronic physical illnesses [40, 41]. Further research will be needed to refine and expand the range of objectives of the INDI model and adapt it to the management of instances of complexity and comorbidity.

We anticipate that with this implementation experiment we will see substantial changes in the care process for depression in primary care. Further, we expect the implementation and generalization of the program will have a global impact, which may be highly relevant considering the presence and repercussions of depression on society and its association with suicidal behaviors.

## Abbreviations

ARCHO: Assessment of Readiness for Chronicity in Health Care Organizations (in Spanish: IEMAC, Instrumento de Evaluación de Modelos de Atención ante la Cronicidad)
CPCQ: Change Process Capability Questionnaire
DSM-V: Diagnostic and Statistical Manual of Mental Disorders, fifth edition
eCAP: Primary Care Clinical Station (in Catalan: Estació Clínica d’Atenció Primària, i. e., the computerized platform of primary care clinical records)
GAF: Global Assessment of Functioning
ICS: Catalan Health Institute (in Catalan: Institut Català de la Salut)
IDIAP: Primary Care Research Institute (in Catalan: Institut d’Investigació en Atenció Primària)
IEXPAC: Chronic Patient Experience Evaluation Tool (in Spanish: Instrumento de Evaluación de la Experiencia del Paciente Crónico)
INDI: Interventions for Depression Improvement
INNÒBICS: Open Research at the Catalan Health Institute (in Catalan: Innovació Oberta a l’ICS)
MINI: Mini International Neuropsychiatric Interview
PACIC: Patient Assessment of Care for Chronic Conditions
PARIHS: Promoting Action on Research Implementation in Health Services
PERIS: Strategic Plan for Research and Innovation in Health (in Catalan: Pla Estratègic de Recerca i Innovació en Salut)
PHQ-9: Patient Health Questionnaire, 9 items
QALY: Quality Adjusted Life Year

## References

[1] Gabilondo A, Rojas-Farreras S, Vilagut G, Haro JM, Fernández A, Pinto-Meza A (2010). Epidemiology of major depressive episode in a southern European country: results from the ESEMeD-Spain project. J Affect Disord.

[2] Sabes-Figuera R, Knapp M, Bendeck M, Mompart-Penina A, Salvador-Carulla L (2012). The local burden of emotional disorders. An analysis based on a large health survey in Catalonia (Spain). Gac Sanit.

[3] Salvador-Carulla L, Bendeck M, Fernández A, Alberti C, Sabes-Figuera R, Molina C (2011). Costs of depression in Catalonia (Spain). J Affect Disord.

[4] Üstün TB, Sartorius N, eds. Mental Illness in General Health Care: An International Study. Chichester: Wiley; 1995.

[5] Aragonès E, Piñol JL, Labad A, Masdéu RM, Pino M, Cervera J (2004). Prevalence and determinants of depressive disorders in primary care practice in Spain. Int J Psychiatry Med.

[6] World Health Organization. Integrating mental health into primary care: a global perspective. Geneva: World Health Organization; 2008.

[7] Fernández A, Pinto-Meza A, Bellón JA, Roura-Poch P, Haro JM, Autonell J (2010). Is major depression adequately diagnosed and treated by general practitioners? Results from an epidemiological study. Gen Hosp Psychiatry.

[8] Wittchen HU, Holsboer F, Jacobi F (2001). Met and unmet needs in the management of depressive disorder in the community and primary care: the size and breadth of the problem. J Clin Psychiatry.

[9] Pinto-Meza A, Fernandez A, Serrano-Blanco A, Haro JM (2008). Adequacy of antidepressant treatment in Spanish primary care. Psychiatr Serv.

[10] Kohn R, Saxena S, Levav I, Saraceno B (2004). The treatment gap in mental health care. Bull World Health Organ.

[11] Gilbody S, Bower P, Fletcher J, Richards D, Sutton AJ (2006). Collaborative care for depression: a cumulative meta-analysis and review of longer-term outcomes. Arch Intern Med.

[12] Gilbody S, Gilbody S, Bower P (2011). Collaborative care. Depression in primary care.

[13] Coventry PA, Hudson JL, Kontopantelis E, Archer J, Richards DA, Gilbody S (2014). Characteristics of effective collaborative care for treatment of depression: a systematic review and meta-regression of 74 randomised controlled trials. PLoS One.

[14] Thota AB, Sipe TA, Byard GJ, Zometa CS, Hahn RA, McKnight-Eily LR (2012). Collaborative care to improve the management of depressive disorders: a community guide systematic review and meta-analysis. Am J Prev Med.

[15] Community Preventive Services Task Force (2012). Recommendation from the community preventive services task force for use of collaborative care for the management of depressive disorders. Am J Prev Med.

[16] Sighinolfi C, Nespeca C, Menchetti M, Levantesi P, Belvederi Murri M, Berardi D (2014). Collaborative care for depression in European countries: a systematic review and meta-analysis. J Psychosom Res.

[17] Aragonès E, Piñol JL, Caballero A, López-Cortacans G, Casaus P, Hernandez JM (2012). Effectiveness of a multi-component programme for managing depression in primary care: a cluster randomized trial. The INDI project. J Affect Dis.

[18] Solberg LI, Crain AL, Jaeckels N, Ohnsorg KA, Margolis KL, Beck A (2013). The DIAMOND initiative: implementing collaborative care for depression in 75 primary care clinics. Implement Sci.

[19] Eccles MP, Armstrong D, Baker R, Cleary K, Davies H, Davies S (2009). An implementation research agenda. Implement Sci.

[20] Aragonès E, Caballero A, Piñol JL, López-Cortacans G, Badia W, Hernández JM (2007). Assessment of an enhanced program for depression management in primary care: a cluster randomized controlled trial. The INDI project (Interventions for Depression Improvement). BMC Public Health.

[21] Aragonès E, Caballero A, Piñol JL, López-Cortacans G (2014). Persistence in the long term of the effects of a collaborative care programme for depression in primary care. J Affect Dis.

[22] Aragonès E, López-Cortacans G, Sánchez-Iriso E, Piñol JL, Caballero A, Salvador-Carulla L, Cabasés J (2014). Cost-effectiveness analysis of a collaborative care programme for depression in primary care. J Affect Disord.

[23] Working Group of the Clinical Practice Guideline on the Management of Depression in Adults. Clinical Practice Guideline on the Management of Depression in Adults. Ministry of Health, Social Services and Equality. Galician Agency for Health Technology Assessment (avalia-t); 2014. SNS Clinical Practice Guidelines: Avalia-t 2013/06.

[24] Adaptació al model sanitari català de la guia de pràctica sobre el maneig de la depressió major en l’adult [Adaptation to the Catalan health model the practice guideline on the management of major depression in adults]. Departament de Salut. Generalitat de Catalunya 2010. Available at: http://aquas.gencat.cat/web/.content/minisite/aquas/publicacions/2010/pdf/adaptacio_gpc_depressio_aiaqs_2010ca.pdf. Accessed 10 Dec 2017.

[25] Aragonès E, Comín E, Cavero M, Pérez V, Molina C, Palao D (2017). Un sistema informatizado de apoyo a las decisiones clínicas para el manejo de la depresión en atención primaria. Aten Primaria.

[26] Cavero M, Monreal JA, Cardoner N, Moreno MD, Pérez-Solá V, Palao D (2017). Efficacy of an active implementation process of a computerized CPG of major depression disorder in primary care. Eur Psychiatry.

[27] Stetler CB, Damschroder LJ, Helfrich CD, Hagedorn HJ (2011). A guide for applying a revised version of the PARIHS framework for implementation. Implement Sci.

[28] Nilsen P (2015). Making sense of implementation theories, models and frameworks. Implement Sci.

[29] Nuño-Solinís R, Fernández-Cano P, Mira-Solves JJ, Toro-Polanco N, Contel JC, Mora MG (2013). Desarrollo de IEMAC, un Instrumento para la Evaluación de Modelos de Atención ante la Cronicidad. Gac Sanit.

[30] Solberg LI, Asche SE, Margolis KL, Whitebird RR (2008). Measuring an organization's ability to manage change: the change process capability questionnaire and its use for improving depression care. Am J Med Qual.

[31] Kitzinger J (1995). Qualitative research. Introducing focus groups. BMJ.

[32] Mira J, Nuño-Solinís R, Guilabert-Mora M, Solas-Gaspar O, Fernández-Cano P, González-Mestre MA (2016). Development and validation of an instrument for assessing patient experience of chronic illness care. Int J Integrated Care.

[33] Glasgow RE, Wagner EH, Schaefer J, Mahoney LD, Reid RJ, Greene SM (2005). Development and validation of the patient assessment of chronic illness care (PACIC). Med Care.

[34] Rossom RC, Solberg LI, Vazquez-Benitez G, Crain AL, Beck A, Whitebird R (2016). The effects of patient-centered depression care on patient satisfaction and depression remission. Fam Pract.

[35] Bolíbar B, Fina F, Morros R, Garcia MD, Hermosilla E, Ramos R, Rosell M (2012). SIDIAP database: electronic clinical records in primary care as a source of information for epidemiologic research. Med Clin (Barc).

[36] Thygesen LC, Ersbøll AK (2014). When the entire population is the sample: strengths and limitations in register-based epidemiology. Eur J Epidemiol.

[37] Murray E, Treweek S, Pope C, MacFarlane A, Ballini L, Dowrick C (2010). Normalisation process theory: a framework for developing, evaluating and implementing complex interventions. BMC Med.

[38] Nock MK, Borges G, Bromet EJ, Alonso J, Angermeyer M, Beautrais A (2008). Cross-national prevalence and risk factors for suicidal ideation, plans and attempts. Br J Psychiatry.

[39] Serrano-Blanco A, Palao DJ, Luciano JV, Pinto-Meza A, Luján L, Fernández A (2010). Prevalence of mental disorders in primary care: results from the diagnosis and treatment of mental disorders in primary care study (DASMAP). Soc Psychiatry Psychiatr Epidemiol.

[40] Gili M, Comas A, García-García M, Monzón S, Antoni SB, Roca M (2010). Comorbidity between common mental disorders and chronic somatic diseases in primary care patients. Gen Hosp Psychiatry.

[41] Aragonès E, Piñol JL, Labad A (2007). Depression and physical comorbidity in primary care. J Psychosom Res.

